# Docosahexaenoic Acid Rescues Synaptogenesis Impairment and Long-Term Memory Deficits Caused by Postnatal Multiple Sevoflurane Exposures

**DOI:** 10.1155/2016/4062579

**Published:** 2016-08-11

**Authors:** Guorong Tao, Yan Luo, Qingsheng Xue, Guohui Li, Yongchang Tan, Jinglei Xiao, Buwei Yu

**Affiliations:** ^1^Department of Anesthesiology, Ruijin Hospital, Shanghai Jiaotong University School of Medicine, Shanghai 200025, China; ^2^Department of Anesthesiology, Xinhua Hospital, Shanghai Jiaotong University School of Medicine, Shanghai 200025, China

## Abstract

Sevoflurane exposures were demonstrated to induce neurotoxicity in the developing brain in both human and animal studies. However, there is no effective approach to reverse it. The present study aimed to evaluate the feasibility of utilizing docosahexaenoic acid (DHA) to prevent sevoflurane-induced neurotoxicity. P6 (postnatal 6 days) mice were administrated DHA after exposure to 3% sevoflurane for two hours daily in three consecutive days. Molecular expressions of synaptic makers (PSD95, synaptophysin) and synaptic morphological changes were investigated by Western blot analysis and transmission electron microscopy, respectively. Meanwhile, Morris water maze test was used to assess spatial memory of mice at P31 (postnatal 31 days). DHA restored sevoflurane-induced decreased level of PSD95 and synaptophysin expressions and increased PSD areas and also improved long-term spatial memory. These results suggest that DHA could rescue synaptogenesis impairment and long-term memory deficits in postnatal caused by multiple sevoflurane exposures.

## 1. Introduction

Previous retrospective studies suggested that children, who had undergone multiple anesthesia and surgeries at an earlier age, were more likely to develop learning disability in future [1, 2]. The studies from neonatal rodents or primates also demonstrated that a majority of general anesthetics had the potential to induce neurotoxicity in the developing brain [3, 4]. Sevoflurane, one of inhalational anesthetics, is commonly used in pediatric anesthesia for its better tolerance and excellent anesthetic effects. Multiple sevoflurane exposures, during brain development period, impaired synaptogenesis and cause severe cognitive decline [5, 6].

Despite extensive studies about the mechanisms by which sevoflurane induced developmental neurotoxicity, there were very rare researches on the methods to reverse it. Docosahexaenoic acid (DHA), a physiologically important omega-3 (*ω*-3) long chain polyunsaturated fatty acid (LC-PUFA), is a structural component for synaptosomal membrane, synaptic vesicles, and growth cones [7]. During embryonic and postnatal period, DHA plays an important role in the neural development [8]. Also, in some pathological conditions, DHA was proved to display good neuroprotective effect [9]. The present study investigates whether DHA rectifies the synaptogenesis impairment and long-term memory deficits in postnatal caused by multiple sevoflurane exposures.

## 2. Methods and Materials

### 2.1. Animals

The animal treatment protocol was approved by the Ethics Committee for the Care and Use of Laboratory Animals of Shanghai Jiao Tong University. C57BL/6 mother mice and their postnatal day 6 (P6) pups were acquired from SLAC Laboratory Animal Co., Ltd. (Shanghai, China). All mice were housed at constant temperature (22°C) under a 12 : 12 h light-dark cycle with* ad libitum* access to food and water; the pups were reared by their mother mice. A total of 13 litters including 88 P6 mice were used in this study. To minimize the difference among different mother mice, we divided the same number of mice from one litter into each experiment group as far as possible. None of mice died in the whole process of anesthesia.

### 2.2. Anesthesia

P6 mice (both genders) were anesthetized with either 3% sevoflurane balanced with 60% oxygen or only 60% oxygen as described in the previous study [5]. The treatment was applied for two hours daily in three consecutive days. During anesthesia, the temperature of anesthesia chamber was maintained at 37.0 ± 0.5°C using a heating plate. DHA (15 *μ*g/g, ultrasonic emulsification in saline) (Sigema, USA) or the same volume of saline was administered intraperitoneally, 30 minutes before sevoflurane or oxygen delivery. The dose of DHA is based on previous study [10] and our preliminary experiment.

### 2.3. Western Blot Analysis

Western blot analysis was performed to detect the effects of sevoflurane on expressions of synapse makers PSD95 and synaptophysin in the hippocampus as described previously [11]. Hippocampus tissues from both hemispheres were harvested after the last sevoflurane exposure and lysed in ice-cold RIPA buffer. The proteins were isolated using SDS-PAGE method and electrophoretically transferred into polyvinylidene difluoride membranes (Millipore, Billerica, MA, USA). The membranes were incubated with primary rabbit anti-PSD95, anti-synaptophysin (1 : 1,000, Cell Signaling, Danvers, MA, USA), or mouse anti-*β*-actin (1 : 5,000, Sigma, St. Louis, MO, USA) antibodies with gentle agitation overnight at 4°C. The membranes were then incubated with horseradish peroxidase-labeled goat anti-rabbit (1 : 2,000) or goat anti-mouse (1 : 5,000) secondary antibodies for 1 hr at room temperature. The protein bands were visualized using ImageQuant LAS 4000 Mini system (GE Healthcare Bio-Sciences, Pittsburgh, PA, USA). The signal intensity of bands was analyzed using Image J software.

### 2.4. Morris Water Maze Test

Morris water maze (MWM) test was performed as described previously with some modifications [5]. Briefly, the P31 mice were trained in a round water-filled steel tank that contained a hidden 10 cm diameter platform. The place trials were performed four times daily for seven successive days. For each trial, the mice were allowed to find the platform for 90 s and were permitted to remain on the platform for 15 s after finding it. Otherwise, the mice were gently guided to the platform and permitted to remain there for 15 s. Escape latencies were recorded to evaluate changes in spatial learning. For each mouse, the probe trial was performed 24 hrs after finishing the last place trial on the seventh day. During the probe trial, the mice were allowed to swim for 90 s after removing them from the platform. Platform crossing times were recorded to evaluate changes in spatial memory for each group.

### 2.5. Transmission Electron Microscopy

About 1 mm^3^ hippocampus tissues were dissected from P8 mice (after three times of treatment) and fixed in 2.0% glutaraldehyde for 2 hrs at 4°C. The tissues were rinsed in 0.1 M cacodylate buffer and postfixed in 1% OsO_4_ for 2 hrs and then dehydrated by a series of graded ethanol. Then infiltration was done by first 1 : 1 resin : acetone for 1 hr, 2 : 1 resin : acetone for 1 hr, pure resin for 1 hr, and finally pure resin overnight. After that, the hippocampus tissues were embedded in a 60°C oven for 48 hrs. 70% ethanol was saturated with uranylacetate to enhance the contrast.

The hippocampus tissues were embedded in EMBED-812 EMBEDDING KIT (Electron Microscopy Sciences). Ultrathin sections (70–80 nm) were cut by Ultracut ultramicrotome (Leica UC6, Germany), mounted on pioloform-coated 50 mesh grids, and contrasted with 0.5% lead citrate for 5 mins. Ultrastructural changes of hippocampus synapses were observed and examined under a transmission electron microscope (PHLIP-CM-120, Holland). The total number of mice for transmission electron microscopy was twelve and the number of each group was three.

### 2.6. Statistical Analysis

Data from Western blot analyses, place trials of the MWM tests, and the area of PSD in TEM were expressed as the means ± SEM. Platform crossing times obtained during the probe trials of the MWM tests were expressed as medians and interquartile ranges. Differences in protein expression levels and the area of PSD between-group or among-group were analyzed using student's *t*-test or one-way ANOVA followed by* post hoc *Newman-Keuls. Two-way repeated measures ANOVA analyses were used to compare the difference of learning curves (based on escape latency) among groups in the MWM place trials.* Post hoc *Bonferroni tests were used to compare the difference in escape latency among groups in each day of MWM place trials. The Mann-Whitney test was used to calculate differences in platform crossing time among groups. *P* values of less than 0.05 (*∗*), 0.01 (*∗∗*), and 0.001 (*∗∗∗*) were considered statistically significant. Statistical analyses were performed using SPSS 18.0 software (SPSS Inc., Chicago, IL, USA). All graphs were plotted using PRISM 5 software (GraphPad, La Jolla, CA, USA) and Adobe Illustrator Artwork 16.0 software (Adobe Inc., San Jose, California, USA).

## 3. Results

### 3.1. Multiple Sevoflurane Exposures Impaired Synaptogenesis in the Hippocampus

**Figure 1:**
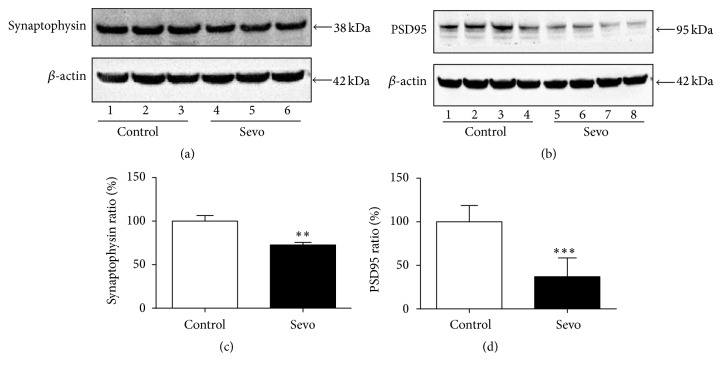
Multiple sevoflurane exposures decreased PSD95 and synaptophysin expressions in the hippocampus. (a) After multiple sevoflurane treatment, PSD95 and *β*-actin expressions in the hippocampus were detected by Western blot analysis. (b) The expressions of synaptophysin and *β*-actin were also determined after anesthesia. (c) The histogram shows PSD95/*β*-actin ratio in (a). (d) The histogram shows synaptophysin/*β*-actin ratio in (b). All data are presented as mean ± SEM; ^*∗∗*^ *P* < 0.01 and ^*∗∗∗*^ *P* < 0.001 comparing with control group, *n* = 6 for each group. Sevo = sevoflurane.

First of all, we investigated whether multiple sevoflurane exposures impaired the synaptogenesis in the developing brain and detected expressions of synapse makers PSD95 and synaptophysin in the hippocampus after three exposures of 3% sevoflurane. Sevoflurane decreased expressions of PSD95 and synaptophysin in the hippocampus tissues (Figures 1(a) and 1(b)). There were significant differences between sevoflurane group and control group (Figure 1(c), *P* = 0.0020; Figure 1(d), *P* = 0.0009). These results indicated that multiple sevoflurane exposures induced synaptogenesis impairment in the hippocampus.

### 3.2. DHA Restored Sevoflurane-Induced Synaptogenesis Impairment

**Figure 2:**
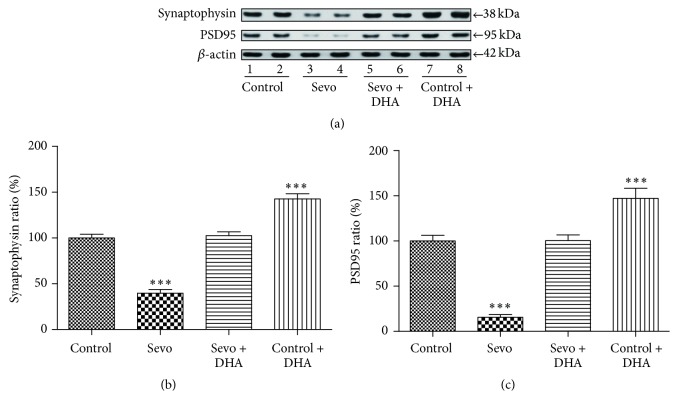
DHA attenuated sevoflurane-induced decreases in the PSD95 and synaptophysin expressions. (a) DHA (15 *μ*g/g) was administered intraperitoneally 30 mins before each treatment with 3% sevoflurane for 2 h in three consecutive days. Immediately after the last exposure, the expression levels of PSD95, synaptophysin, and *β*-actin in the hippocampus tissues of mice were determined by Western blot analysis. (b) The histogram shows the levels of synaptophysin/*β*-actin ratio in (a). (c) The histogram shows the levels of PSD95/*β*-actin ratio in (a). All data are presented as mean ± SEM; ^*∗∗∗*^ *P* < 0.001 comparing with control group, *n* = 6 for each group. Sevo = sevoflurane.

To determine the neuroprotective effects of DHA, we performed DHA pretreatment before sevoflurane anesthesia and then investigated the synaptogenesis performance.  Figure 2(a) showed the expressions of PSD95, synaptophysin, and *β*-actin in the control, Sevo (sevoflurane), Sevo + DHA, and control + DHA groups. DHA can rescue the decreasing of SYP [*F*(3, 20) = 86.14; *P* < 0.0001] and PSD95 [*F*(3, 20) = 56.28; *P* < 0.0001] after applying multiple sevoflurane anesthesia (Figures 2(b) and 2(c)), which suggested that DHA had potential effects to rescue synaptogenesis impairment after multiple sevoflurane anesthesia.

### 3.3. DHA Mitigated Sevoflurane-Induced Synaptic Ultrastructure Injuries

**Figure 3:**
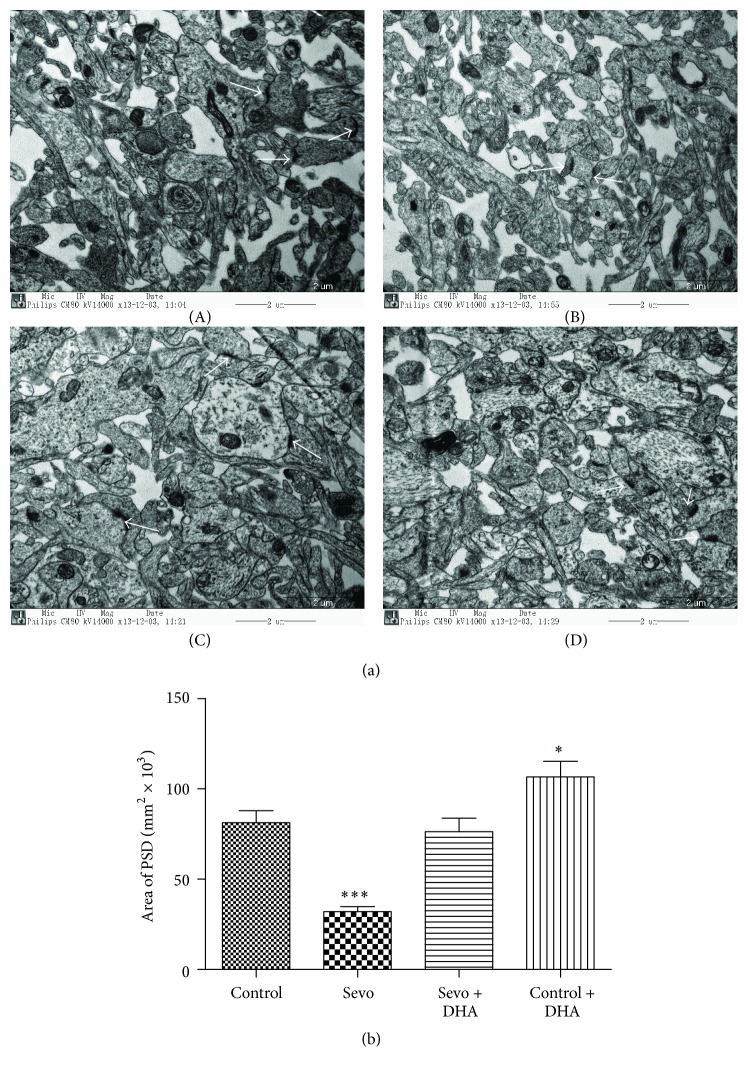
DHA alleviated synaptic ultrastructure impairment after sevoflurane exposure. (a) Hippocampal synaptic ultrastructure in the control group ((a)(A)), Sevo group ((a)(B)), Sevo + DHA group ((a)(C)), and control + DHA group ((a)(D)) was analyzed by transmission electron microscopy. White arrows showed postsynaptic densities (PSD). Scale bar = 2 *μ*m. (b) Quantitation of PSD areas was represented. Data are shown as mean ± SEM; ^*∗∗∗*^ *P* < 0.001 and ^*∗*^ *P* < 0.05 comparing with control group, *n* = 9 for each group. Sevo = sevoflurane.

To confirm the neuroprotective effects of DHA, we further investigated the change of synaptic ultrastructure by transmission electron microscopy. Three pictures of each mouse were analysed, so nine pictures of each group were analysed. DHA can rescue the decreasing PSD (white arrow) [*F*(3, 32) = 20.91; *P* < 0.0001] after applying multiple sevoflurane anesthesia (Figures 3(a) and 3(b)), which was consistent with the molecular PSD95 and synaptophysin performances in Figure 2. PSD in the Sevo group were significantly smaller than that in the control group (Figures 3(a)(A), 3(a)(B), and 3(b) 32.05 ± 8.42 versus 82.35 ± 19.89, *P* < 0.0001). However, DHA pretreatment attenuated sevoflurane-induced reduction of PSD areas (Figures 3(a)(C), 3(a)(A), and 3(b) 76.23 ± 22.64 versus 82.35 ± 19.89, *P* > 0.05). DHA alone can increase the PSD areas (Figures 3(a)(D), 3(a)(A), and 3(b) 106.70 ± 26.07 versus 82.35 ± 19.89, *P* < 0.05).

### 3.4. DHA Attenuated Long-Term Memory Deficits Caused by Multiple Sevoflurane Exposures during the Postnatal Period

**Figure 4:**
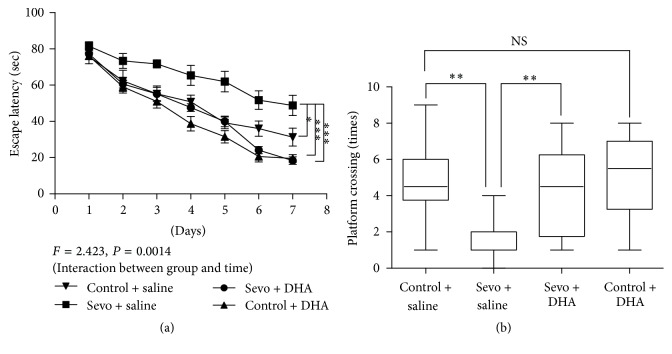
DHA pretreatment improved spatial memory performance in the adulthood. (a) The escape latency of control + saline group, Sevo + saline group, Sevo + DHA group, and control + DHA group in the place trials of MWM was recorded in the seven successive days. (b) The platform crossing times in control + saline group, Sevo + saline group, Sevo + DHA group, and control + DHA group were shown. The data in (a) are presented as mean ± SEM. The data in (b) are shown as median and 95% confidence interval. ^*∗*^ *P* < 0.05, ^*∗∗*^ *P* < 0.001, and ^*∗∗∗*^ *P* < 0.001 comparing with control group or sevoflurane group, *n* = 10 in each group. MWM = Morris water maze. Sevo = sevoflurane. NS = no significance.

Previous studies demonstrated that sevoflurane exposure inhibited hippocampal synaptic plasticity, long-term learning, and memory [6, 12]. Therefore, we investigated whether DHA pretreatment could ameliorate sevoflurane-induced long-term cognition decline. As shown in Figure 4(a), there was a statistically significant interaction of time and treatment in terms of escape latency of place trials among four groups (two-way ANOVA with repeated measurement, *P* = 0.0014).* Post hoc *Bonferroni test showed that mice in control group, Sevo + DHA group, and control + DHA group had faster escape latency than those in Sevo + saline group at P33, P34, P35, P36, and P37 (*P* < 0.05, *P* < 0.01, or *P* < 0.001) (Figure 4(a)). Furthermore, DHA attenuated sevoflurane-induced decreases in platform crossing times during the probe trials, which indicated memory improvement after DHA intervention [*F*(3, 36) = 6.106, *P* = 0.0018]. And the platform crossing times in Sevo + saline group were significantly shorter than those in control + saline group (*P* < 0.01) and Sevo + DHA group (*P* < 0.01). However, there was no significant difference in the platform crossing times between control + saline group and control + DHA group (*P* > 0.05). These results from MWM test suggested that DHA might exhibit protective effects against sevoflurane-induced long-term cognition deficits.

## 4. Discussion

In the present study, we found that anesthesia with 3% sevoflurane, for two hours daily from P6 to P8, decreased the PSD areas, expressions of synaptophysin, and PSD95 in the hippocampus. Meanwhile, spatial memory performance in the adulthood decreased after multiple sevoflurane exposures. However, DHA, one of *ω*-3 polyunsaturated fatty acids, could not only restore sevoflurane-induced synaptic morphologic and molecular depressions but also improve long-term memory performance, which indicated that DHA could rescue synaptogenesis impairment and long-term memory deficits caused by multiple sevoflurane exposures in postnatal.

Consistent with other previous studies [6, 11, 12], three times of sevoflurane anesthesia decreased the PSD95 and synaptophysin expressions in hippocampus. PSD95 and synaptophysin were postsynaptic and presynaptic markers, respectively, and its reduction meant synaptic loss. Meanwhile, synaptic ultrastructure analysis showed that PSD areas significantly decreased after multiple sevoflurane exposures. During the spurt of brain growth, synapse formation constituted the most important incident. Distribution of synaptogenesis may cause serious consequences. Our study showed that repeated sevoflurane exposures induced memory deficits in the adulthood.

The most important finding was that DHA showed a good prospect to prevent sevoflurane-induced synaptogenesis impairment and long-term memory deficits. DHA, as a basic structural component of membrane phospholipids, is enriched in brain [13]. Under physical condition, DHA plays an important role in the brain development and memory formation. DHA deficits may hamper neurite growth for neural development [14] and decrease soma size of hippocampus neuron and number of synaptic vesicles after learning task [15, 16]. We showed that DHA supplementation could reverse sevoflurane-induced synaptic injuries and improve the memory performance in the adulthood, which were consistent with other studies showing neuroprotective effects of DHA [17, 18].

DHA may promote synaptogenesis through diverse mechanisms. First of all, DHA provided precursors for phosphatides that were essential for establishing synaptic membranes [19, 20]. Furthermore, DHA could promote translation of synapse-related genes and increased total levels of synaptic proteins [20]. A recent study also reported that DHA had capabilities to increase BDNF synthesis in the developing brain and helped form synaptic connectivity [21]. Whether DHA rescued sevoflurane-induced synaptogenesis impairment through the mechanisms above needed to be clarified in the following experiments. In addition, our and other previous studies showed that sevoflurane exposure in the developing brain induced significant levels of neuroapoptosis and neuroinflammation, which may reduce number of neurons for forming synapses [11, 22]. In fact, recent studies showed that DHA supplementation could prevent neuronal apoptosis via decreasing caspase-3 and Bax expressions [23]. Also DHA had potent effects against neuroinflammation in various models [24, 25]. Whether DHA ameliorated sevoflurane-induced synaptogenesis through inhibiting neuroapoptosis and neuroinflammation was an interesting question and also needed to be elucidated in future.

In conclusion, multiple exposures of 3% sevoflurane might inhibit synaptogenesis in the developing brain and cause spatial memory deficits when grown up. DHA pretreatment could rescue sevoflurane-induced synaptogenesis impairment and long-term cognitive decline.
